# Modelling white matter with spherical deconvolution: How and why?

**DOI:** 10.1002/nbm.3945

**Published:** 2018-08-16

**Authors:** Flavio Dell'Acqua, J.‐Donald Tournier

**Affiliations:** ^1^ Institute of Psychiatry Psychology and Neuroscience, King's College London Department of Neuroimaging UK; ^2^ Sackler Institute for Translational Neurodevelopment, Institute of Psychiatry Psychology and Neuroscience, King's College London Department of Forensic and Neurodevelopmental Sciences UK; ^3^ King's College London Division of Imaging Sciences and Biomedical Engineering UK

**Keywords:** diffusion imaging, diffusion tensor imaging, fiber orientation density function, fiber response, ODF, MRI, spherical deconvolution, tractography

## Abstract

Since the realization that diffusion MRI can probe the microstructural organization and orientation of biological tissue *in vivo* and non‐invasively, a multitude of diffusion imaging methods have been developed and applied to study the living human brain. Diffusion tensor imaging was the first model to be widely adopted in clinical and neuroscience research, but it was also clear from the beginning that it suffered from limitations when mapping complex configurations, such as crossing fibres. In this review, we highlight the main steps that have led the field of diffusion imaging to move from the tensor model to the adoption of diffusion and fibre orientation density functions as a more effective way to describe the complexity of white matter organization within each brain voxel. Among several techniques, spherical deconvolution has emerged today as one of the main approaches to model multiple fibre orientations and for tractography applications. Here we illustrate the main concepts and the reasoning behind this technique, as well as the latest developments in the field. The final part of this review provides practical guidelines and recommendations on how to set up processing and acquisition protocols suitable for spherical deconvolution.

**Abbreviations used**
AFD: apparent fibre density
CHARMED: composite hindered and restricted model of diffusion
CSF: cerebrospinal fluid
CSD: constrained spherical deconvolution
CX: structural complexity
DTI: Diffusion tensor imaging
DSI: diffusion spectrum imaging
dODF: diffusion orientation density function
DPI: diffusion propagator imaging
dRL‐SD: damped Richardson Lucy algorithm
fODF: fibre orientation density function
FOD: fibre orientation distribution
FD: fibre density
FS: fibre spread
HARDI: high angular resolution diffusion imaging
HMOA: hindrance modulated orientational anisotropy
MAP: mean apparent propagator
MCMC: Markov chain Monte Carlo
NuFO: number of fibre orientations
NPA: narrow pulse approximation
QBI: Q‐ball imaging
RL‐SD: Richardson‐Lucy spherical deconvolution
SHORE: simple harmonic oscillator based reconstruction and estimation
SPFI: spherical polar Fourier imaging

## 1 INTRODUCTION

Even before diffusion MRI was proposed as a method for tractography,^1^, ^2^, ^3^ it was recognized that the diffusion tensor model was strongly affected by the presence of crossing fibres. In their influential 1996 publication, Basser and Pierpaoli noted that their proposed measures of anisotropy were highly dependent on the degree of coherence of fibre tract directions.^4^ Nevertheless, the unique ability to extract orientational information non‐invasively from living biological tissues made the diffusion tensor one of the main tool of modern neuroimaging.^5^ Many tensor‐based tractography methods have since been proposed and successfully used in clinical and neuroscience research applications.^6^, ^7^, ^8^, ^9^

**Figure 1:**
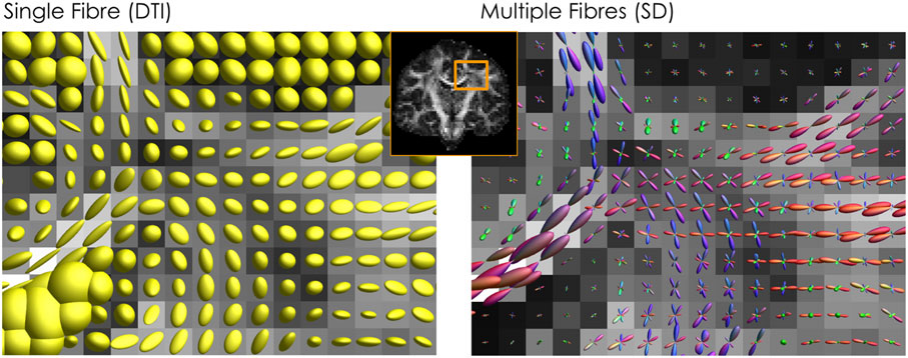
Modelling the diffusion signal. On the left, diffusion ellipsoids from the diffusion tensor model describe the average diffusion profile within a voxel and provide information for a single dominant fibre orientation. On the right, by adopting a multi‐fibre approach, such as spherical deconvolution, multiple fibre orientations can be identified and visualized using fODFs

**Figure 2:**
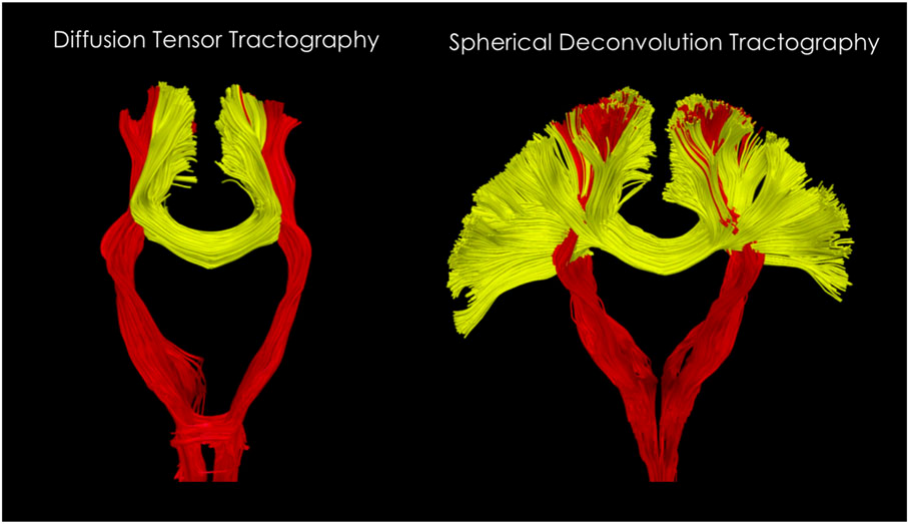
Diffusion tensor and spherical deconvolution tractography. On the left, the diffusion tensor can be used to reconstruct major white matter pathways but only describing the average fibre orientation within each voxel and not resolving crossing fibres. In this example, lateral projections of the corpus callosum are not reconstructed because they are interrupted by the more dense and dominant components of the corticospinal tract. On the right, by using multi‐fibre methods it is possible to track through the corticospinal tract and visualize a much larger portion of the corpus callosum

**Figure 3:**
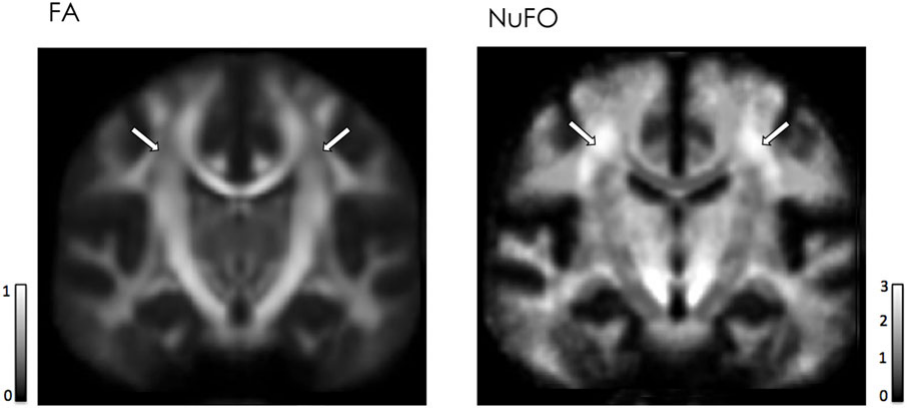
Effect of fibre crossing on diffusion metrics. On the left, regions of low FA value are evident within the centrum semiovale. On the right, the same regions correspond to areas with high numbers of fibres crossing (≥3) as shown by the number of fibre orientations (NuFO) map

However, while these early successes contributed to a fast adoption of these techniques in several research fields, the limitations of the tensor model started to become evident when larger sections of the clinical research community, unaware of the main limitations of the tensor model, started to adopt tractography and tensor‐derived metrics for many different applications. Three clear examples of these limitations are the inability: (i) to resolve and visualize crossing configuration between white matter tracts using DTI (Figure 1); (ii) to correctly delineate and track white matter pathways that traverse regions of crossing fibres (Figure 2); and (iii) to differentiate between fibre coherence and intrinsic white matter properties using tensor‐derived metrics (Figure 3). This last point, in particular, has led to the widespread and often misleading use of terms such as ‘white matter integrity’ as a synonym for fractional anisotropy, even when differences or changes in this metrics are often only driven by different degrees of fibre coherence.^10^, ^11^, ^12^

It took a further decade before the full impact of crossing fibres on tractography and tensor‐derived metrics became broadly recognized,^11^, ^12^, ^13^, ^14^, ^15^, ^16^ especially within the non‐technical clinical and neuroscience communities. There are probably many reasons why the issue of crossing fibres was not appreciated earlier. One reason is the lack of viable alternatives. While the idea of performing 6D *q*‐space imaging experiments to map the full diffusion propagator, and potentially resolve complex microstructural tissue organization, had already been proposed by Paul Callaghan and co‐workers in 1988,^17^ the first measurement of crossing fibres was only demonstrated using diffusion spectrum imaging (DSI) in 2000.^18^ However, this technique was not viable at the time due to its unrealistic scan time requirements. A number of other techniques were proposed over the next few years: multi‐tensor fitting,^19^ PAS‐MRI,^20^ *Q*‐ball imaging,^21^ spherical deconvolution,^22^, ^23^ ball and sticks,^13^ and modern constrained variants of spherical deconvolution,^24^, ^25^, ^26^, ^27^ to name but a few. Only with these last few methods has it become feasible to robustly process data acquired using clinically feasible protocols.

Furthermore, while these methods provided improved fibre orientations for use in tractography, many studies still remained reliant on the diffusion tensor model to provide ‘microstructural’ measures^28^, ^29^ (e.g. fractional anisotropy, radial and axial diffusivities) due to the lack of alternatives. Indeed, many of these new fibre orientation estimation methods relied on the fundamental assumption that white matter fibres did not differ in their diffusion properties between the different tracts (see ‘fibre response function’ below for details), implying that any observed differences in tensor‐derived measures of ‘microstructure’ were purely driven by partial volume effects (e.g. crossing fibres). This assumption was entirely at odds with the prevailing view that diffusion MRI could be used to report on microstructure and ‘white matter integrity’. Consequently, many researchers elected to keep using tensor‐derived measures in their studies, albeit with a much more cautious and better informed interpretation of any differences observed.

A further reason for the slow adoption of these methods was an under‐appreciation of the true extent of these issues within the technical community. Initially the problem of crossing fibres was felt by most researchers to be restricted to a few problematic areas (particularly the pons and centrum semiovale), with the bulk of the white matter believed to be broadly unaffected. This vision has now evolved since more studies have consistently shown that crossing is indeed a common configuration within most of white matter. Recent estimates suggest that between 70% and 90% of the entire white matter in the human brain is characterized by at least two or more fibre populations crossing within the same region.^13^, ^16^, ^30^, ^31^ This makes crossing a main feature of essentially all white matter voxels, and it cannot be ignored when performing tractography or extracting microstructure metrics.

Several methods are available today to deal with the ‘crossing fibre problem’, and different diffusion models or approaches can be organized in precise families and categories. In this review, we outline the principles behind these methods, describe the main techniques proposed in the literature, focussing particularly on spherical deconvolution approaches, and discuss current trends in the field.

## 2 APPROACHES TO FIBRE ORIENTATION ESTIMATION

The problem of fibre orientation estimation has been the focus of very active research, and has been approached from a number of different perspectives. Two main camps can clearly be discerned: methods based on *q*‐space, and methods based on mixture models. These are sometimes referred to as ‘model‐free’ and ‘model‐based’ approaches respectively—although as we discuss later, these labels are somewhat misleading. Methods based on *q*‐space typically estimate the so‐called *diffusion orientation density function* (dODF), while methods based on mixture models typically estimate the *fibre orientation density function* (fODF). This is an important distinction, discussed in detail below.

Methods also differ in the type of output they provide, with ‘parametric’ approaches providing estimates of the various parameters of a discrete model (e.g. the orientations and volume fractions of the fibre populations identified), and ‘non‐parametric’ approaches providing estimates of a continuous distribution of orientations: e.g. the fibre orientation distribution (FOD), *aka* the fODF. However, these labels are not always clear‐cut, since some methods represent a continuous fODF using ‘parametric’ models (e.g. a mixture of Watson distributions). Nonetheless, this is a useful classification, since this often makes a big difference in the optimization methods needed to solve the problem, and consequently the computational requirements. Most parametric models allow for a variable number of fibre orientations, which can introduce issues due to the need for a model selection strategy before or during the actual model fitting. Furthermore, selecting a different number of model parameters across different brain voxels is likely to introduce an uneven stability in the model fitting with respect to MR noise.

### 2.1 *Q*‐space

A full description of the *q*‐space formalism is available from other articles in this special issue, and will not be repeated here.^17^, ^32^, ^33^ However, it is helpful to discuss those aspects that relate specifically to fibre orientation estimation. The central concept in *q*‐space is the Fourier relationship between the *ensemble spin propagator* *P*(***r***, Δ), evaluated at a particular diffusion time Δ and displacement ***r***, and the diffusion‐weighted signal *S*(*q*, Δ) acquired as a function of the *q*‐vector ***q*** ∝ ***G****δ*, with ***G*** and *δ* corresponding to the diffusion‐encoding gradient vector and its duration respectively. The aim of most *q*‐space‐based methods is to recover an estimate of the propagator *P*(***r***, Δ), or at least its angular dependence in the particular case of fibre orientation estimation. This angular dependence is typically encapsulated in the dODF, defined as the radial projection of the ensemble spin propagator $\Psi \left(\hat{u},\Delta \right)=\int_{0}^{\infty }P\left(r\hat{u},\Delta \right)\;r^{2}\;dr$, with $\hat{u}$ the unit vector corresponding to the direction of displacement, and *r* its scalar magnitude. Peaks in the dODF are then assumed to correspond to fibre orientations, since these are the directions of highest spin displacement.

Methods based on *q*‐space are often thought of as ‘model free’, since *q*‐space itself assumes no model. While this may be true for the propagator, and by extension the dODF, this no longer applies when the dODF is used as a *fibre* orientation estimator. While the dODF is clearly strongly dependent on the fibre orientations, it is nonetheless distinct. Hence, to extract fibre orientations from the dODF, a model needs to be assumed. The most common approach is to simply use the peaks in the dODF directly as estimates of the fibre directions. However, this has been shown to introduce bias when the fibres cross at non‐orthogonal angles.^34^, ^35^ More advanced methods have been proposed, notably the *Q*‐ball sharpening transform,^31^ which interestingly reduces to a spherical deconvolution (see later).

Another notable concern with methods based on *q*‐space is the fact that the narrow pulse approximation (NPA) simply cannot be met in practice. To satisfy this criterion, the diffusion gradient pulses should be applied for a time so short that the diffusion of water molecules is negligible, at least compared with the axonal structures of interest. Using the Einstein equation, this implies that sub‐millisecond pulse durations are needed if the root‐mean‐square displacement of water molecules is to remain smaller than the typical axonal radius of 1 μm—something that would require gradient strengths orders of magnitude larger than currently achievable, with commensurate slew rates. However, for the purposes of fibre orientation estimation, violation of the NPA is likely to actually be beneficial, by improving the contrast in the angular domain.^36^ This should therefore not be viewed as a major concern here.

A number of methods have been proposed based on *q*‐space. Diffusion spectrum imaging (DSI)^18^ was the first such technique, originally demonstrated in 2000.^37^ DSI is essentially the direct application of *q*‐space to imaging, requiring full sampling of the diffusion encoding gradient vector space. However, its onerous acquisition requirements have limited its adoption, and in practice the estimated dODF suffers from reconstruction artefacts even with a relatively large number of *q*‐space samples (e.g. ~500 DWI volumes).^38^, ^39^ A number of modifications have been proposed since, to allow for non‐Cartesian acquisitions and/or improve the robustness of the reconstruction.^38^, ^39^, ^40^, ^41^, ^42^, ^43^

*Q*‐ball imaging (QBI) was proposed shortly after DSI as a means of estimating the dODF directly from data acquired using the much more time‐efficient high angular resolution diffusion imaging (HARDI) protocol.^21^, ^44^ Rather than acquire data on a regular Cartesian grid in *q*‐space, data were instead acquired over a dense set of directions at a constant *q*‐value (or equivalently *b*‐value). QBI relies on the Funk‐Radon transform to estimate the dODF, avoiding the need to the full Fourier transform. However, this provides an approximation to the true dODF that does not include the *r*^2^ term in the radial projection, leading to reduced angular resolution compared with the dODF obtained by DSI. Nonetheless, QBI was widely adopted due to its much more relaxed acquisition requirements and fast reconstruction. A number of modifications have been proposed since, including the use of spherical harmonics to speed up and improve the reconstruction,^45^, ^46^ its extension to multi‐shell data,^41^ and solid‐angle considerations to provide a dODF equivalent to that provided by DSI.^47^

A number of other approaches based on *q*‐space have also been proposed over the years, including persistent angular structure MRI (PAS‐MRI),^20^ diffusion orientation transform,^48^ generalized diffusion tensor imaging,^49^, ^50^ and many others. However, these are less commonly used, and in some cases are not developed specifically to estimate the directions of the fibres themselves, but rather are used to characterize the diffusion signal in the presence of crossing fibres. More recently, thanks to improvements in data acquisition strategies and the ability to collect significantly more MR diffusion data within reasonable scan times, there has been renewed interested in developing methods based on *q*‐space to better characterize propagator metrics and improve dODF reconstruction. Methods such as simple harmonic oscillator based reconstruction and estimation (SHORE),^51^ mean apparent propagator (MAP)‐MRI,^52^ spherical polar Fourier imaging (SPFI)^53^ and diffusion propagator imaging (DPI)^54^ are all examples of a growing family of methods based on the decomposition of the diffusion signal as a linear combination of different functional bases.

### 2.2 Mixture models

In contrast to *q*‐space methods, mixture or multi‐compartmental models attempt to estimate the fibre orientations and their volume fractions directly, by assuming a particular *model* for the signal that would be measured for a single fibre population. In these methods, the signal measured within a voxel is assumed to correspond to the sum of the DW signals that would have been measured for each fibre population in isolation (or from distinct compartments, e.g. CSF)—in other words, *exchange* is assumed to have a negligible effect. Some of these methods also include other compartments in the model, such as an isotropic signal to account for partial volume with CSF or grey matter. The many methods that have been proposed to date differ with respect to the signal model assumed for the fibres, the inclusion of other compartments, the parameterization used to represent the orientation information, the inclusion of regularization or constraints, and the particular algorithm used to solve the problem.

Of these, the parameterization used to represent the orientation information is arguably the most important conceptually. Many approaches assume that a discrete number of fibre populations are present with well‐defined orientations, leading to a compact but variable number of parameters, typically requiring at least three parameters per fibre orientation (two for the *θ*, *φ* orientation, one for volume fraction).^13^, ^19^, ^55^, ^56^, ^57^, ^58^, ^59^, ^60^, ^61^, ^62^ In contrast, other approaches represent the orientation information as a continuous fibre ODF (*aka* FOD). This is parameterized using a fixed, large number of coefficients, for example spherical harmonics,^22^, ^23^, ^44^, ^45^, ^46^, ^63^, ^64^, ^65^, ^66^ or dictionary‐based approaches.^20^, ^21^, ^24^, ^25^, ^26^, ^67^ There are also approaches that combine elements of the continuous and discrete representations: these model the fODF as a discrete set of fibre populations, each with its own amount of dispersion, leading to a continuous fODF.^68^, ^69^, ^70^, ^71^, ^72^, ^73^ These various representations are discussed in more detail in a subsequent section.

The signal originating from each fibre population can be modelled using the diffusion tensor model or a simplified version of it, e.g. an axial symmetric tensor (where second and third eigenvalues are assumed to be equal) or totally anisotropic tensor (where second and third eigenvalues are set to zero, *aka* ‘stick’ model). This leads directly to multi‐tensor approaches, whereby the signal is assumed to be the weighted sum of the signals from individual tensors. Given an independent estimate of the number of fibre populations present, the relevant parameters can be estimated using non‐linear fitting routines,^19^ or Markov chain Monte Carlo (MCMC) sampling approaches.^13^, ^58^ The simpler the model used for each fibre population, the larger the number of distinct fibre populations that can typically be extracted using multi‐tensor fitting approaches. It is worth mentioning that by assuming the same axial symmetric tensor or the same stick model across all fibre populations the problem reduces to a spherical deconvolution, as will be described in more detail in the following sections.

A more sophisticated approach is to assume also different types of diffusion within different compartments. For example, in the composite hindered and restricted model of diffusion (CHARMED), diffusion within axons is described using analytical models of restricted diffusion in impermeable cylinders.^55^ However, this and other similar models are more related to the field of microstructure imaging than to the actual problem of resolving and estimating multiple fibre orientations. We will not provide a detailed description of microstructure models here, but refer the reader to the corresponding reviews^74^, ^75^ from this special issue.

### 2.3 Diffusion ODF or fibre ODF?

While many methods have been proposed to analyse diffusion MRI data, there is still no clear consensus about what the best approach is. In this section, we discuss the issues that are involved, and outline our particular point of view.

**Figure 4:**
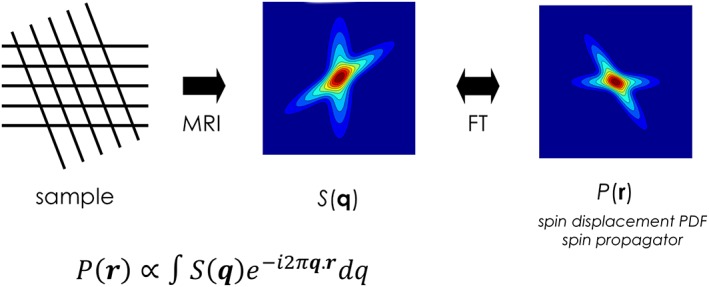
MR diffusion signal and diffusion propagator: by acquiring the diffusion signal with regular *q*‐space sampling, the diffusion propagator can be directly obtained through Fourier transformation of the MR signal

**Figure 5:**
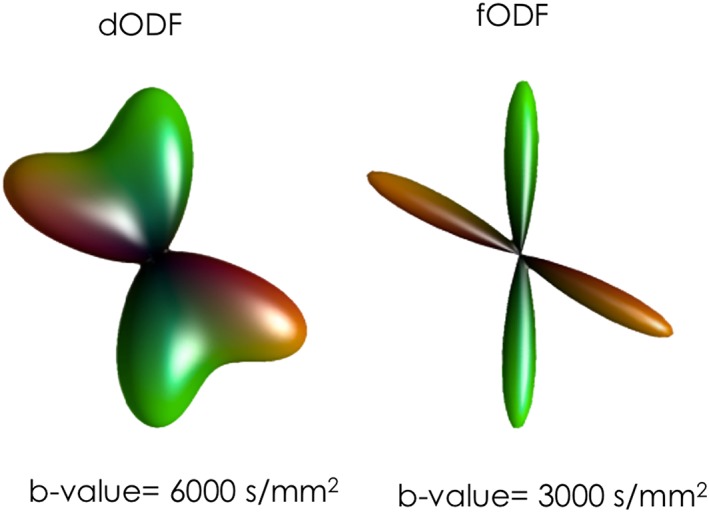
60° fibre crossing configuration visualized using dODF and fODF profiles. On the left, the dODF is obtained from DSI, with a maximum *b*‐value of 6000 s/mm^2^. On the right, the fODF is obtained using spherical deconvolution with *b* = 3000 s/mm^2^

The clearest point of divergence between the different methods is whether they attempt to estimate the *diffusion* ODF via the ensemble‐averaged spin propagator, relying on the theory of *q*‐space (illustrated in Figure 4), or instead aim to recover the *fibre* ODF directly, in this case typically relying on a mixture model. Proponents of the dODF argue that it remains the most unbiased, model‐free characterization of the physical process of diffusion itself: any attempt at extracting more specific information (in particular the fODF) relies on assumptions about how tissue microstructure influences the MRI signal. On the other hand, proponents of the fODF argue that the dODF is only providing an indirect and blurred representation of the underlying fibre orientation. Even in the presence of a single coherently oriented fibre population, the dODF will provide a smooth profile, suggesting not a single sharp fibre population but rather a broad range of possible orientations centred about the main orientation. Since the diffusion propagator is a smooth function, the computation of the dODF is inevitably penalized by an intrinsic angular blurring that limits angular resolution or the ability to resolve two distinct fibre populations. In contrast, an fODF can return a more accurate description for a single or multiple fibre populations by directly ‘modelling out’ (or deconvolving, in the case of deconvolution methods) the angular blurring introduced by the diffusion process and recovering more precisely the information of fibre orientation. For a single coherently oriented fibre population, the fODF can be represented by a delta function oriented along the direction of the fibre population. In practice, however, due to regularization procedures and to account for noise instabilities and numerical errors, a smoother fODF is always preferred.^30^ Nevertheless, as shown in Figure 5, the recent literature shows that fODF methods consistently produce sharper functions compared with methods based on Dodf.^11^, ^31^, ^34^, ^76^

The requirement for a precise characterization of the FOD becomes critical in applications such as tractography, where the estimated parameters are then used for further processing. In tractography, the fibre‐tracking algorithm attempts to follow the estimated *fibre* orientations.^1^, ^3^, ^13^, ^76^, ^77^, ^78^, ^79^ Given these algorithms' explicit requirement for fibre orientation estimates, it makes sense to use methods that estimate the fODF directly, rather than the dODF. From a different point of view, while it may be possible to estimate the dODF in a model‐free manner, this information cannot be used for tractography directly without assuming an appropriate model. Most dODF‐based approaches to tractography will simply use the directions of the peaks in the dODF as estimates of the fibre orientations^80^, ^81^, ^82^, ^83^, ^84^; this in many ways can be viewed as a model, although not a particularly sophisticated one, leading to significant inaccuracies when the crossing angle is less than 90°.^34^, ^35^ While more sophisticated approaches can be devised to estimate the fODF from the dODF,^31^, ^85^ it is noteworthy that the model introduced to achieve this is in essence identical to that used in methods that do estimate the fODF directly.

Moreover, tractography is not the only context that relies on accurate estimation of the fODF. New microstructure models have been devised that attempt to extract microstructural information from crossing fibre voxels,^55^ or voxels with a variable amount of dispersion.^71^ Given the tight interaction between the microstructure parameters (e.g. axon diameter distribution, fibre density) and the fODF,^30^, ^86^ it is important that the fODF is estimated as accurately as possible if the microstructure parameters are to be reliably found.

For these reasons, we believe that methods that estimate the fODF have proved to be more useful in practical situations. This is not to say that dODF estimates are not valuable; quite the opposite: in many cases they can still provide reliable model‐free information when pathological changes cannot be fully modelled, and they can also be used to effectively identify differences across subjects. However, *interpreting* such changes in biological terms will by necessity require a model of the microstructure and the pathology under investigation.

### 2.4 Continuous representation of the orientation information

As mentioned previously, methods differ in the way in which the fibre orientation information is represented. The continuous distribution has the advantage of generality: with this approach it is possible to represent any arrangement of fibre orientations within a given voxel, allowing issues such as fibre dispersion, curvature or ‘fanning’ to be taken into account.^87^, ^88^ The spherical harmonic basis is commonly used,^22^, ^23^, ^44^, ^45^, ^46^, ^63^, ^64^, ^65^, ^66^ as well as dictionary‐based approaches.^20^, ^21^, ^24^, ^25^, ^26^, ^67^ These continuous representations have the additional advantage of linearity with the signal, which brings considerable advantages in terms of computational efficiency; this allows for the use of very fast linear methods, leading to reconstruction times of the order of seconds to minutes for typical whole‐brain data.

These different approaches each come with their own pros and cons. For instance, the spherical harmonic basis, being the spherical equivalent of the Fourier series, has compelling mathematical properties, including notably an elegant convolution theorem,^89^ but suffers from issues such as the spherical equivalent of Gibbs ringing. Dictionary‐based approaches approximate the function as the weighted sum of direction‐specific kernels, where each kernel may correspond to the angular point spread function (e.g. sRBF),^21^, ^67^ or the signal that would be expected for a fibre population aligned along that direction.^25^, ^26^ This representation has the advantage that constraints such as non‐negativity and sparsity can be efficiently imposed directly on the coefficients (i.e. the weights). However, they also typically require several hundred directions for faithful fODF reconstruction,^21^, ^25^, ^26^ leading to an under‐determined problem that is then solved by imposing strong constraints such non‐negativity or sparsity (see below).

### 2.5 Discrete representation of the orientation information

The fibre orientation information can also be represented as a discrete set of fibre populations (referred to in recent work as *fixels*: a fibre population within a voxel^90^, ^91^). The advantage of such approaches is that of parsimony: each fixel is typically described by few parameters (e.g. orientation (*θ*, *ϕ*) and volume fraction (*f*)), and the number of fixels per voxels is assumed to be low.^13^, ^55^, ^58^, ^62^ Most of these approaches also include an isotropic fast diffusing compartment to model cerebrospinal fluid (CSF). Some approaches attempt to estimate more parameters per fixel than others, for example a full six‐parameter tensor,^19^ an axially symmetric tensor^55^, ^56^, ^57^, ^59^, ^60^ or measures of dispersion of the fibres about the main orientation of the fibre population.^68^, ^69^, ^70^, ^71^, ^72^, ^92^ Note that we include in this group approaches that provide a continuous approximation to the fODF (as opposed to a set of delta functions), by allowing each fibre population to have its own orientation dispersion; this blurs the distinction between the fixel‐based and continuous representations, by allowing these methods to represent more general fibre configurations while preserving the parsimony of fixel‐based models.

There are two main disadvantages to these representations. First, the relationship between fibre orientations and signal is not linear: non‐linear fitting methods are therefore required to solve for the parameters, leading to long reconstruction times, and requiring great care to ensure convergence to the global minimum and stable solutions. Second, the number of fixels included in the fit will typically vary depending on the evidence provided by the data, and this can strongly affect the estimated parameters and the stability of the fit with respect to noise. The number of fixels is typically determined using model comparison techniques, such as an *F*‐test,^63^, ^93^ using the Bayes factor^57^ or automatic relevance determination.^13^ However, in noisy data these methods will consistently favour simpler models, for example by assuming a single fibre population when three are present in reality (see,e.g., Reference 13, Appendix A). In such situations, the results will be consistently biased: the estimated orientations will be wrong if the wrong number of directions has been used. This could also mean that the orientations estimated in one voxel may not be consistent with those in neighbouring voxels, simply because the number of fixels has been incorrectly determined. This has obvious implications for applications such as tractography, but also more generally for microstructure modelling, where a reliable estimate of the number and direction of the fibre populations is critical.^94^

### 2.6 Solving the inverse problem

Diffusion MRI data is typically noisy, and the inverse problems that need to be solved are typically very poorly conditioned. Thankfully, there are a number of constraints and regularizers that can be applied to obtain stable solutions that are very robust to noise.

#### 2.6.1 Linear constraints

Linear regularizers can be used, such as minimum norm (which helps reduce instabilities due to non‐uniformly distributed DW gradient directions), low pass filtering (which helps to attenuate high frequency noise)^23^ and Laplace‐Beltrami regularization.^45^ While these can already improve the quality of results, they typically result in a loss of angular resolution, due to the strong attenuation of the high angular frequency components that these methods typically introduce.

#### 2.6.2 Non‐negativity and sparsity constraints

Under the right circumstances, powerful non‐linear regularizers or constraints can also be applied. Two of the most commonly employed include non‐negativity^25^, ^26^, ^27^ and sparsity.^26^ However, these can only meaningfully be applied on the fODF, based on the knowledge that the fODF must be positive semi‐definite (since it represents densities), and the expectation that voxels will typically contain a small number of well‐defined orientations. Of these, non‐negativity is arguably the most powerful, is already inherently sparsity promoting and is always a safe assumption (there is no biologically plausible situation where the fibre density can be negative). On the other hand, sparsity constraints may introduce biases in cases where significant dispersion or curvature is expected, since they may tend to collapse what should be a relatively broad distribution into a single well‐defined peak. They can also lead to biases if applied too strongly, by suppressing small, yet non‐negligible, secondary fibre orientations. Note that both constraints are also implicit in most fODF estimation methods that use the discrete representation (see above), since the parameterization is inherently sparse, and volume fractions are typically constrained to be positive.

#### 2.6.3 Unit integral constraint

**Figure 6:**
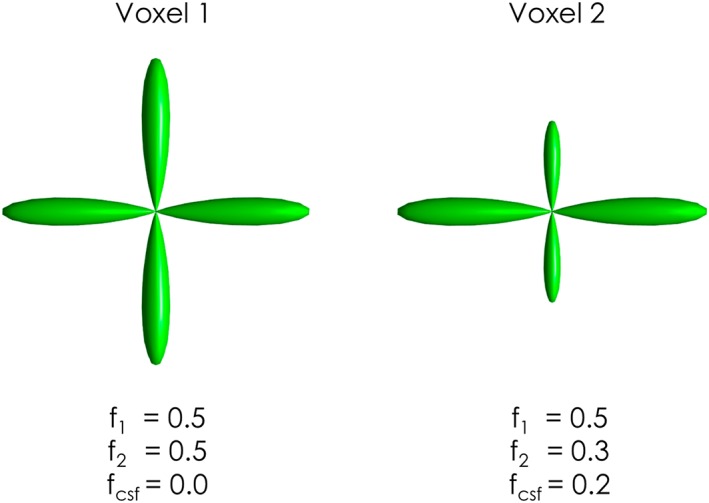
Violation of the fODF unit integral constraint. One the left, a two‐fibre crossing configuration (volume fractions *f*_1_ = 0.5, *f*_2_ = 0.5). On the right, the same two‐fibre crossing configuration with additional CSF partial volume (volume fractions *f*_1_ = 0.5, *f*_2_ = 0.3, *f*_csf_ = 0.2). By comparing the two fODFs it is easy to verify that the two fODF profiles cannot be constrained to have the same (unit) integral while also preserving the same amplitude for the lobes with *f* = 0.5

Another constraint that is sometimes applied is that of unit ODF integral, motivated by the interpretation of the ODF as a probability density function. However, this is equivalent to forcing the fODF to have a constant average value. Sadly, the fODF cannot be assumed to be normalized, since it is directly proportional to the DW signal (whether normalized to the *b* = 0 signal or not), which is itself not constant (Figure 6). For instance, if we consider Voxel A containing pure white matter, and Voxel B containing a mixture of white matter and CSF, it can be seen immediately that Voxel B will have reduced DW signal because, for any reasonable *b*‐value, the CSF signal will be strongly attenuated and the voxel signal will be dominated by the white matter fraction. This difference is further exaggerated when considering the DW signal normalized to its *b* = 0 signal: due to the longer *T*_2_ of the CSF fraction, Voxel B will also have a much larger *b* = 0 signal, further increasing the difference with Voxel A. Enforcing unit integral therefore is not correct and does not make sense given the nature of the data, and the linear relationship between signal and fODF that most methods implicitly or explicitly assume.

Note that this constraint is similar to the one used in most fibre orientation and diffusion microstructure estimators that assume a discrete representation of the signal (see above), where the sum of the volume fractions for all compartments modelled is set to unity. However, these models typically also include explicit CSF and other compartments, allowing the volume fractions of the fibre populations to sum up to less than unity, correctly reflecting the actual biophysical heterogeneity of different voxels. Our criticism of the unit integral constraint therefore only applies to methods that enforce the unit integral for the fibre compartments only.

#### 2.6.4 Neighbourhood smoothness constraints

Given that the fODF represents the directions of white matter pathways projecting over relatively long distances, it makes sense to use this information to help regularize the estimation of the fODF. While it is possible to simply use an isotropic smoothing kernel to filter the ODF in the spatial domain, this will inevitably introduce a loss of spatial resolution. However, it is fair to assume smoothness *along* the fibre pathways, making the use of anisotropic smoothing and total variation spatial regularization techniques appealing,^95^, ^96^ whereby smoothness is enforced along peaks in the fODF, but not across them. This concept is elegantly captured in the fibre continuity approach,^97^ with clear advantages for resolving crossing fibres. While this type of approach can be particularly powerful for the analysis of data consisting of very few directions,^98^ it can also lead to problematic results with very low quality data when the orientation information content is simply too low, allowing the smoothness prior to dominate. In such cases, a spatial regularization approach can provide fODF results that look superficially clean, but are clearly biased and paradoxically lead to inferior tractography results. Directly related to spatial regularization, a further benefit of using neighbourhood information is the possibility of estimating asymmetric fODF rather than traditional symmetric fODF.^99^, ^100^ Although these methods are still in their infancy and not yet widely applied, if they prove to be successful to better discriminate complex configurations such as bending, crossing and kissing fibres in real data they could lead to a substantial improvement in existing tractography methods.

## 3 SPHERICAL DECONVOLUTION

Due to the large number of possible approaches to estimating fibre orientations, it is impossible to review them all in detail. However, based on the issues discussed above, we believe that a good approach to fibre orientation estimation should.

- estimate the *fibre‐ODF*, rather than the *diffusion*‐ODF:
  ○ in practice, almost all applications will need fibre orientations as input;
  ○ it allows more powerful constraints to be used;
  ○ model‐free methods are no longer model‐free by the time *ad hoc* approaches are used to extract the fibre orientations;
- use a continuous representation of the ODF:
  ○ it can be used to represent any fibre configuration;
  ○ it does not rely on model selection methods to choose a discrete number of fibre populations;
  ○ it allows for the use of fast, linear solvers;
- impose a non‐negativity constraint, whether implicitly or explicitly;
- *not* impose a unit integral constraint.

Based on the above considerations, the spherical deconvolution approach is a natural choice. For this reason, the rest of this review will focus on this class of methods and discuss issues specific to its various implementations.

**Figure 7:**
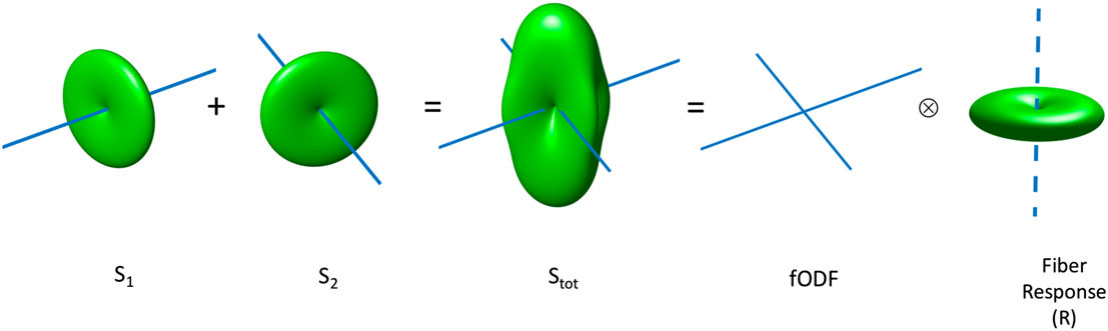
An illustration of the spherical convolution approach: multiple fibre populations within a voxel contribute with additive signals (*S*_1_, *S*_2_) to the total DW signal (*S*_tot_). Under the assumption of a common fibre signal profile, this is equivalent to the convolution over the sphere of an fODF with a chosen fibre response function (*R*)

The central concept behind spherical deconvolution is that the DW signal measured for any fibre population is sufficiently similar that any differences can to all intents and purposes be ignored. The DW signal will always be low along the main axis of the fibres, and relatively preserved across the fibres. Provided with a good estimate of the DW signal for a canonical fibre population, the problem can be expressed as a linear sum of the signals for all the fibre populations present in a given voxel (Figure 7). When these fibre populations are represented in terms of a more general *distribution* of fibre orientations (*aka* the fODF), this mixture of signals becomes a *spherical convolution*. The problem of estimating the fibre orientations themselves is then solved by inverting the problem, to infer the fODF from the measured signal given a suitably calibrated response for a canonical fibre population.

A number of approaches have been proposed to date based on this general concept. They differ in a number of respects:

- how the response function is derived;
- what constraints are applied;
- how the fODF is represented;
- the methods used to solve the inverse problem.

In the following sections, we focus on two implementations in current use, constrained spherical deconvolution (CSD) and Richardson‐Lucy spherical deconvolution (RL‐SD), and discuss issues common to most methods.

### 3.1 Constrained spherical deconvolution (CSD)

This approach uses the spherical harmonic basis to represent the fODF, and applies a non‐negativity constraint as a soft regularizer.^27^ Briefly, the algorithm solves the problem

$$\hat{f}=\min_{f}\;{\left\|\mathrm{Hf}-m\right\|}^{2}+\lambda {\left\|\mathrm{Af}\right\|}_{-}^{2}.$$

The coefficients *f* of the fODF are estimated as those that minimize the least‐squares fit of the predicted signal to the data *m* at the voxel of interest, while also minimizing the sum of squares of the negative amplitudes in the fODF. The predicted DWI signal is obtained via the matrix *H* = *MR*, where *M* maps SH coefficients to amplitudes along the DW directions sampled, and *R* is a diagonal matrix of response function coefficients performing the spherical convolution in the spherical harmonic domain.^89^ The matrix *A* maps SH coefficients to a dense set of uniformly distributed directions, and the ${\left\|x\right\|}_{-}^{2}$ operator computes the squared norm of the *negative* components of *x*. The algorithm typically converges within 5–10 iterations per voxel, leading to whole‐brain computation times of the order of 10 s on a modern desktop computer.

The spherical deconvolution problem being solved is in itself relatively straightforward, and shares many similarities with other spherical deconvolution approaches.^26^ However, the implementation of CSD as proposed differs from others in three main respects. First, the solution is by no means guaranteed to be non‐negative: non‐negativity is imposed as a soft regularizer, penalizing negative values. While this may seem sub‐optimal, there are some advantages to this approach: (i) it is computationally very efficient; and (ii) the use of the SH basis means that some degree of Gibbs ringing is to be expected; while this could be removed using a hard constraint, this will inevitably lead to some loss in angular resolution. The second difference is the use of an empirically determined response function, rather than one derived from a specific model (see below for further details). Third, the deconvolution is performed on the raw DW signal without prior normalization to the *b* = 0 signal. While this could be argued to compromise quantification, the use of an empirically determined, subject‐specific response counters this to a large extent (in other words, the response function is implicitly scaled in proportion to the corresponding DWI data, so that gross differences in scaling will be factored out in the deconvolution). More importantly, operating on the raw DW signal preserves the linearity of the fODF to the DW signal even in the presence of CSF (or any other tissue with large differences in *T*_2_), providing more robust estimates of fibre density, particularly when using moderate to high *b*‐values.

### 3.2 Richardson‐Lucy spherical deconvolution (RL‐SD)

A well‐known deconvolution algorithm in the field of image restoration and inverse problems is the Richardson‐Lucy (RL) algorithm.^101^, ^102^ Originally developed to restore astronomical images, the RL algorithm can be considered a particular case of the expectation maximization algorithm^103^, ^104^ where the Bayesian framework is applied to the deconvolution problem in the case of Poisson noise.^105^ Over the years, RL algorithms have been modified to include more noise distributions, including Gaussian^106^ and Rician^95^ noise, while also accommodating several regularization strategies.^105^, ^107^, ^108^ The main features of the RL algorithm are its robustness both to noise and to inaccuracies in the definition of the fibre response (or point spread function), and its implicit enforcement of a non‐negative constraint,^30^, ^105^, ^109^ important requirements for the specific diffusion problem. A first implementation of the RL algorithm for spherical deconvolution for Gaussian noise distribution can be described as^25^

$${\left[f^{\left(k+1\right)}\right]}_{i}={\left[f^{\left(k\right)}\right]}_{i}\frac{{\left[H^{T}s\right]}_{i}}{{\left[H^{T}Hf^{\left(k\right)}\right]}_{i}}$$

where *k* is the *k* ^th^ algorithm iteration; [**f**]_*i*_ is the *i* ^th^ element of the *n* × 1 vector **f** containing the fODF values calculated along a uniform set of directions (e.g. 752); **s** is the *m* × 1 vector of DWI signals acquired along the HARDI sampling (e.g. 60 DWI directions) normalized to the *b* = 0 signal. Finally, **H** is the *m* × *n* circulant matrix describing the fibre response function, where every column represents the fibre response profile oriented along one of the *n* directions. In this form, the algorithm only requires fast matrix–vector multiplications and element‐wise divisions without any matrix inversion or estimation of derivatives. Also, since **H** and **s** are non‐negative by construction, it easy to verify that non‐negativity of the solution is always guaranteed as long as the initial condition [**f**^(0)^]_*i*_ ≥0 is set. Global convergence to the maximum likelihood solution of the algorithm has been demonstrated by De Pierro.^110^ Moreover, because RL algorithms are also semi‐convergent,^109^, ^111^ the number of algorithm iterations is often used as a practical regularization parameter to control the final smoothness or sharpness of the recovered fODF. While calibration is usually required to identify the optimal trade‐off between angular resolution of the fODF and noise stability, once defined these settings are stable and consistent across subjects and dependent only on the acquisition protocols.

A second and more broadly adopted version of this algorithm is the modified *damped Richardson Lucy* algorithm (dRL‐SD).^24^ By following a regularization approach originally introduced by White,^108^ this version introduces a regularization strategy directly based on the absolute amplitude of the recovered fODF. It is easy to verify that the smaller the amplitude of an fODF lobe the less reliable its actual estimate is, because a smaller portion of the diffusion signal in the voxel is supporting this orientation. Therefore, compared with larger lobes, smaller fODF components require a stronger regularization as they are more likely to be susceptible to noise and partial volume effects from other tissues. A full description and implementation of the dRL‐SD algorithm is given in Reference 24. In practice, the dRL‐SD algorithm is very effective in suppressing spurious fODF lobes or reducing ringing effects to negligible amplitudes in the presence of partial volume with isotropic or pathological tissue.^119^ When applied to tractography, this has led to accurate reconstructions of the major human brain pathways and to an improved ability to describe complex white matter anatomy.^112^, ^113^, ^114^, ^115^ Finally, it is worth mentioning that the discrete nature of the fibre response matrix, **H**, makes RL algorithms essentially a dictionary‐based spherical deconvolution approach. Here, any type of fibre response (either model based or signal sampled) can be assigned, but more importantly this also allows a straightforward extension to multi‐shell data and multi‐tissue deconvolution by simply providing the right dictionary of fibre responses and tissue types in a similar fashion as for the multi‐tissue version of CSD.^116^ In this case, because contributions from other tissues can be separated from the fODF, the requirement for damping partial volume contaminations is also alleviated.

### 3.3 Fibre response function

All spherical deconvolution methods require an estimate of the single‐fibre response function. Ideally, this corresponds to the DW signal that would be acquired for a unit volume of white matter coherently aligned along a single axis. A number of approaches can be used here, including model‐based approaches such as the axial symmetric diffusion tensor model,^13^, ^25^, ^26^, ^57^ a model with a distribution of diffusivities^62^ and direct empirical measurements from selected single‐fibre voxels identified by either explicit thresholding of brain regions with high FA^23^, ^27^ or through iterative calibration of the fibre response.^117^, ^118^ Approaches based on a tensor model are simple to implement and have been shown to offer already, for practical cases, a very close approximation to more complex fibre response models based e.g. on restricted diffusion.^30^ It can be shown that, given a *b*‐value, if *α* = *D*_∥_ − *D*_⊥_, the entire shape of the fibre response signal profile is then controlled by the single parameter *α*.^24^, ^25^ If appropriately calibrated, a model‐based fibre response not only performs well,^119^ but in some SD implementations can effectively be used as an additional regularization parameter to balance between angular resolution and noise stability of the recovered FOD.^24^, ^25^ This also relaxes the need for precise quantification or knowledge of the exact fibre response.

On the other hand, the advantage of an empirically measured response is that it does not rely on any particular model of the white matter, can be used in situations where the specifics of the response function are not known (e.g. for *ex vivo* data) and also incorporates the inherent dispersion in the axon direction that will in practice be observed even in the most coherent fibre bundles^88^, ^120^; whether this inherent ‘jitter’ about the main bundle orientation should be reflected in the response function (leading to sharp fODFs) or not (leading to more dispersed ODFs) is an interesting question that has yet to be resolved.

**Figure 8:**
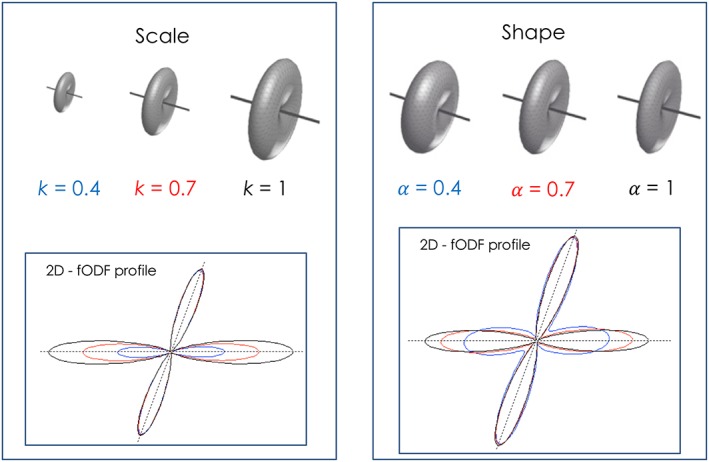
Effect of *scale* and *shape* differences in the fibre signal profile on the final fODF amplitude. If we describe each fibre population with a signal profile $S\left(\theta \right)=e^{-b\left(D_{∥}\cos^{2}\theta +D_{⊥}\sin^{2}\theta \right)}$ we can also express the signal as $S\left(\theta \right)=k\;e^{-\mathrm{b\alpha}\cos^{2}\theta }$ and effectively separate a *scale* parameter $k=e^{-bD_{⊥}}$ and a *shape* parameter *α* = *D*_∥_ − *D*_⊥_. The scale parameter *k* represents the radial hindrance of the fibre acting as a pure scaling factor on the fibre signal and corresponding fODF lobe (left). This term is effectively indistinguishable and equivalent to changes in volume fraction or fibre density. The shape parameter 𝛼 represents a measure of anisotropy between axial and radial diffusivity of the fibre. While small changes in shapes mostly affect only final fODF amplitude, larger shape differences also affect the final shape of the fODF (right) (modified from Reference 30)

A criticism commonly made of spherical deconvolution approaches (and other similar model‐based approaches) is that the response function may not be constant throughout the brain. There is evidence of differences between white matter tracts in their axonal diameter distributions, for instance,^121^ which would be expected to affect the corresponding diffusion signal. However, there are reasons to believe that assuming a fixed fibre response is in fact a relatively benign approximation. First, it is certainly highly beneficial, since the problem then remains linear and can be solved robustly and efficiently. Second, even relatively large changes in axial and radial diffusivities have a relatively small impact on the *shape* of the response, with the dominant effect being on its overall *scale* or amplitude (Figure 8).^30^ This will translate into an incorrect estimate of the fibre density along the corresponding direction, but crucially the orientation estimate itself will be largely unaffected.^23^, ^30^ Another point is that, at moderate to high *b*‐values, the DW signal (and hence the response function) seems to be largely dominated by the intra‐axonal compartment.^71^, ^86^ In these conditions, it is difficult to envisage a situation where only the intra‐axonal compartment would be affected to an extent sufficient to impact its DW signal: this would almost certainly translate to a breakdown in the axonal membrane, implying that the axon is itself no longer viable. A more likely scenario is a reduction in axonal density, with a commensurate increase in the extra‐axonal compartment: while this would most definitely lead to a reduction in anisotropy, this would be mediated primarily by an increase in the *b* = 0 signal and a corresponding reduction in the *average* DWI signal (assuming the signal from the extra‐axonal space is small at moderate to high *b*‐values). Crucially though, this observation of a macroscopic reduction in anisotropy does not imply that the response function is no longer appropriate, but as we will see in the following paragraph this can lead to the introduction of new tract or orientation‐specific metrics, as opposed to more classical voxel (averaged) metrics. Using a healthy response function in this case will provide a result in line with expectation: a reduction in the fODF amplitude, corresponding to a reduction in fibre density. Furthermore, with the existing hardware, it is proving remarkably difficult to measure even relatively large changes in the microstructure (e.g. axonal diameter distributions^122^), implying that these second order effects really do have a relatively small impact on the measured signal. Finally, recent works using spherical tensor encoding suggests that, in white matter, the microstructural anisotropy is remarkably uniform across the brain, lending support to the validity of assuming a constant response function.^123^

Nonetheless, some methods have been proposed to estimate a response function per voxel.^22^, ^124^ This is a difficult problem to solve, given the tight relationship between the fODF and the response function: the signal is inherently smooth, and this smoothness can be captured in either the response or the fODF, without otherwise affecting the quality of the fit. In Reference 124, the response is estimated based on the premise that the fODF is sharp, an assumption that will not in general hold in regions of curvature or dispersion. On the other hand, Reference 22 relies on the observation that, when the response function is modelled as an axially symmetric tensor, the radial diffusivity and mean diffusivity uniquely determine the mean DWI signal (averaged over all orientations) relative to the *b* = 0 signal, irrespective of the fODF (similar to Reference 125). By assuming a fixed mean diffusivity, the radial and axial diffusivities of the response can therefore be computed per voxel, and the resulting response used in the deconvolution. However, the assumption of constant mean diffusivity will break down in regions of partial volume effects with CSF or atrophy, leading to an artificial broadening of the response that is no longer representative of the fibres themselves. This therefore remains a very difficult problem to solve in a way that is generally applicable. Furthermore, this still does not allow for different response functions per fibre population, something that would logically be required if the response function is assumed to differ between different fibre tracts.

## 4 EXTRACTING METRICS FROM MULTIPLE FIBRE ORIENTATIONS

The presence of multiple fibre populations within each imaging voxel complicates the interpretation of traditional scalar measures based on the average tissue properties within a voxel. Using DTI, for example, FA differences may be linked to either real biological changes or just different degrees of fibre dispersion or crossing, and the very definition of axial and radial becomes ill defined when multiple fibre orientations are present.^11^, ^12^ Similarly, most microstructure models today are either explicitly designed around the assumption of a single (coherently or not coherently oriented) fibre or a finite number of coherently oriented bundles of fibres. However, if dispersion and multiple fibre orientations are not considered at the same time these approaches may break down because dispersion will interfere with the estimation of distinct fibre orientations and their properties, and vice versa.

On the other hand, the use of a framework based on the fODF has the advantage of directly incorporating both types of information. This can allow the extraction of new voxel‐wise metrics, not affected by the underling rotational organization, and also tract‐specific or fixel‐wise metrics that only capture bundle‐ or orientation‐specific information within each voxel.

### 4.1 Measures based on rotationally invariant features

Assuming a spherical convolution framework, it can be shown that the mean signal over each *b*‐value shell is independent of the orientations present—it depends exclusively on the mean of the fODF, modulated by the corresponding mean of the response function for this shell. Hence, any measure derived from the mean DW signal per shell will also be invariant to the microstructural rotational organization. This result is used in techniques such as apparent fibre density (AFD): the voxel‐wise mean AFD is given by the mean of the fODF.^86^, ^126^ This idea is also used to derive voxel‐wise measures of the radial and axial diffusivities, based on their relationship with the mean DW signal as a function of *b*‐value in a multi‐shell experiment.^125^ More recent work has extended this further by noting that the fODF can be factored out by taking ratios of the power in the dMRI signal within each spherical harmonic band (i.e. the sum‐of‐squares of the SH coefficients for a given value of the harmonic order *l*) at different *b*‐values, yielding a wider range of rotationally invariant features that can be related to microstructural parameters assuming a simple model of the tissue.^127^ Finally, although not unique to fODF methods, the number of fibre orientations (NuFO) is also an interesting metric that has the potential to offer additional information about the complexity of white matter organization and possibly help to detect longitudinal changes in the presence of white matter degeneration.^13^, ^16^, ^30^, ^31^

### 4.2 Tract‐specific metrics

Since its original definition, it has been clear that fODF profiles not only encode the orientational information of each fibre component, but also can provide useful information about the fibre population itself. Assuming a convolution model to describe the diffusion signal, the absolute amplitude of each fODF can be directly associated with an apparent fibre density (AFD), an index directly ‘related to diffusion properties and density of fibres’^24^ and therefore sensitive to changes of the ‘volume fractions of underlying fibre populations, and to deviations of the actual response function’.^86^ This index can be coupled with a statistical framework allowing more specific fixel‐wise group comparisons instead of traditional voxel‐wise analysis. This is equivalent to comparing the partial volume fractions of fibre populations, allowing for the identification of white matter differences along specific tracts across subjects.^90^, ^91^

An equivalent index based on a slightly different interpretation of the fODF is the hindrance modulated orientational anisotropy (HMOA).^30^ One of the reasons for the different name is to better capture the complex nature of the fODF profile. In fact, differences in fODF amplitude may not only depend on fibre density but also reflect different diffusion properties from distinct fibres. Therefore, even when the assumption of a constant fibre response within a voxel is not met (e.g. in pathological tracts), information from the fODF amplitude can still be considered meaningful. Thus, while HMOA is less specific as a name, it offers a more general and parsimonious term to characterize white matter changes or pathology. Nevertheless, current literature uses AFD and HMOA interchangeably.

A series of similar metrics has been also introduced in Reference 128. Here, AFD is defined instead as angular fibre density (1/(mm^3^rad)), and the fibre density (FD) is the integral of each fODF lobe parametrized as a Bingham distribution. Other metrics are then derived: fibre spread (FS) and structural complexity (CX), describing respectively the broadness of each fODF peak and the complexity of the shape of the whole fODF profile. As discussed in a previous section, describing fibre orientations using Bingham or Watson distributions has been proposed by different authors as a way of quantifying fibre dispersion.^71^, ^92^, ^129^ When applied directly to fODF profiles, the advantage is that it does not require any modification or add more complexity in the actual fODF estimation since Bingham fitting is applied to the final fODF. Fibre dispersion can be quantified for each lobe independently with a fast linear fit,^128^ or with a single non‐linear multi‐Bingham fit to better account for the mixing effect of overlapping fODF lobes.^130^ In conclusion, the fODF profile is very rich in information not easily accessible by traditional voxel‐based metrics, and can therefore offer powerful insights into the underlying microstructural organization of the human brain.

## 5 ACQUISITION CONSIDERATIONS

The many methods available to estimate the full diffusion or fibre ODF each come with their own acquisition recommendations, making it very difficult to implement a future‐proof acquisition protocol likely to be suitable for a wide range of reconstruction methods. Moreover, the increasing availability of multi‐shell acquisition and reconstruction methods, coupled with improved acquisition strategies (notably simultaneous multi‐slice methods^131^), means that single‐shell acquisitions are likely to be gradually phased out and replaced by multi‐shell acquisition in most of future research projects. Nonetheless, there are a few recommendations that can be derived for single‐shell methods, many of which are applicable for multi‐shell approaches.

### 5.1 Optimal *b*‐value

**Figure 9:**
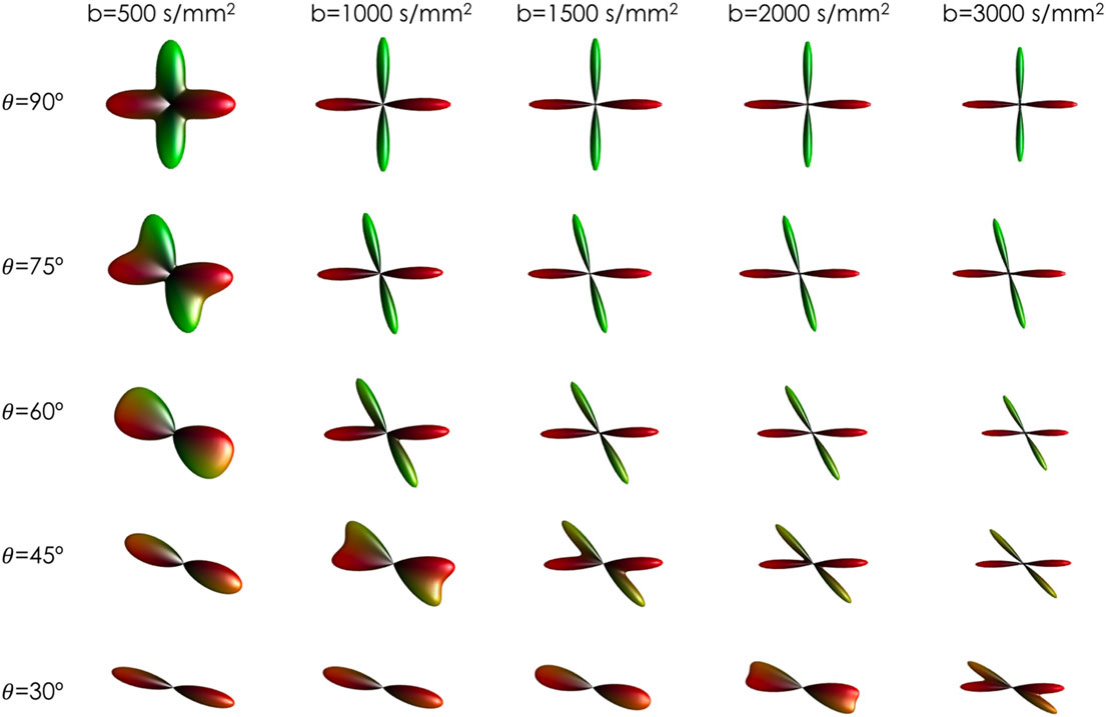
Effect of different b‐values on fODF reconstruction. From left to right, higher *b*‐values give higher angular resolutions and increased ability to detect multiple fibre orientations at smaller angles

The question of what *b*‐value is optimal is still not entirely resolved, although a consensus is gradually emerging, at least for single‐shell HARDI methods. One problem is that this *b*‐value depends in many respects on the target reconstruction. It has been shown previously that *b*‐values in the range of 1/ADC are generally close to optimal for the estimation of tensor‐based measures such as anisotropy and mean diffusivity. However, in terms of fibre orientation estimation, in a crossing fibre context, higher *b*‐values have been shown to provide better angular resolution (Figure 9), with values in the region of 2500–3000 s/mm^2^ consistently coming up as optimal.^118^, ^132^, ^133^

While *b*‐values of this order are readily achievable on modern clinical systems, their use undoubtedly reduces the overall SNR of the images compared with the values more typically used for DTI (typically around *b* = 1000 s/mm^2^). This reduction is partly a consequence of the longer echo times required to accommodate the inevitably longer DW gradients, leading to signal loss through *T*_2_ decay. However, the main reason for the decrease in DW signal is the increase in diffusion *contrast*: while the signal is more strongly attenuated for most DW gradient orientations, it is relatively well preserved for orientations perpendicular to the fibre axis. Hence, while the images may superficially appear to be too noisy to be useful, the overall set of image volumes provides increased contrast to noise ratio and angular resolution to resolve the features in the DW signal that allow fibre orientation estimation.

However, the use of higher *b*‐values can lead to problems with image pre‐processing, notably for eddy‐current and motion correction. Until recently, most approaches for such correction relied on image registration using generic metrics such as correlation ratio or normalized mutual information. These methods can struggle with the lower SNR and increased contrast in the images obtained with higher *b*‐values.^134^ Thankfully, methods now exist that are capable of handling such data with very good results.^135^, ^136^

### 5.2 Number of directions

The optimal number of directions to use in single‐shell HARDI is difficult to ascertain, due to its interaction with SNR, *b*‐value and the specific reconstruction method used, as well as its exact parameters. However, some general recommendations are provided in Reference 130. First, the *b*‐value determines the angular frequency content of the DW signal: the DW signal corresponding to a single coherently oriented fibre population changes from being perfectly isotropic at *b* = 0 to a progressively flatter profile as the *b*‐value increases. To capture these sharper angular features requires sampling along a larger number of DW gradient directions. This means that, while a minimum of 28 DW directions might be sufficient at *b* = 1000 s/mm^2^, 45 DW directions would be required at *b* = 3000 s/mm^2^.^132^

While a minimum number can be identified, using more directions will improve the overall SNR of the reconstruction, simply by contributing more measurements. While different reconstructions will have different SNR requirements, it is generally best to ensure SNR ≥ 15 in the *b* = 0 images (as measured within the brain structures of interest, *not* within CSF), since below this, the signal in the DW images starts to approach the noise floor, introducing problems with Rician bias, which makes any form of averaging problematic. Furthermore, SNR will depend on the *b*‐value (through its effect on the echo time), the resolution of the images, the field strength and the use of partial Fourier or parallel imaging, amongst other parameters. This makes it difficult to provide general recommendations for protocol optimization. However, it is now fairly common to see protocols consisting of 60–90 DW directions at *b* = 2000–3000 s/mm^2^, acquired at 2 mm isotropic resolution, with 10–15 min scan times, being used in routine clinical practice. Similar considerations can also be made for multi‐shell data (see later), where the number of directions in each shell can be decided depending on the maximum angular information available at each *b*‐value. For example, 30, 60 and 90 directions will be more than adequate for a *b* = 500, 1500 or 3000 s/mm^2^ protocol.

## 6 IMPACT AND FUTURE OUTLOOK

With estimation of fibre orientations from single‐shell HARDI data now relatively well established, other avenues of research have opened up further possibilities. While a full review of these latest developments is beyond the scope of this article, it is worth mentioning the most promising ones.

### 6.1 Simultaneous multi‐slice acquisition

While this technique was originally proposed many years ago,^137^ it has only recently become widely adopted for diffusion MRI since the introduction of the blipped CAIPI modification.^131^, ^138^ With this method, it becomes possible to acquire more than one slice per excitation with minimal SNR penalty, allowing for a drastic reduction in scan time, and/or increase in the amount of data collected. For example, by acquiring two slices per excitation (i.e. *multi‐band* factor 2), the scan time can be halved. With higher multi‐band factors, scans that would otherwise have prohibitively long scan times become feasible. However, the corresponding reduction in repetition time reduces the available signal through reduced *T*_1_ relaxation, and the increase in the g‐factor with higher accelerations may amplify the effect of noise in the images. Nonetheless, diffusion data are now routinely acquired with multi‐band acceleration factors between 2 and 3, already offering substantial reductions in scan times. This development has opened the possibility of acquiring data previously confined to research studies: higher spatial resolution, multiple *b*‐values (see *multi‐shell data* below), multiple diffusion times etc. We therefore anticipate that the widespread availability of simultaneous multi‐slice sequences on modern scanners will lead to a step change in the quality and quantity of the data collected, and a corresponding surge in novel reconstruction and modelling algorithms.

### 6.2 Multi‐shell data and microstructure imaging

Data acquired over multiple *b*‐value *shells* are becoming increasingly popular. In these approaches, the diffusion sensitization is applied along uniformly distributed directions for each of a set of distinct *b*‐values. Characterizing the *b*‐value domain in this way opens up new possibilities, for example by allowing for decomposition of the diffusion signal into distinct tissue types, each with its own orientation density function,^116^, ^139^ or the fitting of higher order models.^52^ Disentangling multiple tissue types can also improve the estimation of the fODF itself by removing or modelling out signal contributions not related to fibre orientations, as proposed in recent multi‐shell spherical deconvolution implementations.^116^ Not only this could increase the specificity of tract‐specific metrics and the accuracy of fibre orientations, but it could also help to integrate with microstructure‐specific metrics. Since most microstructure models assume a model of coherently oriented fibres, they are currently adversely affected by crossing fibres or dispersion.^12^, ^73^ Merging fODF with microstructure frameworks could therefore be beneficial for both approaches, leading to more robust diffusion metrics and improved tractography reconstructions. This is currently an area of active research, which we anticipate will lead to many exciting new developments in the near future.

## 7 CONCLUSION

The realization that the microstructural organization and orientation of white matter could be estimated non‐invasively *in vivo* in the human brain using diffusion MRI, and that these orientations could be used for fibre tracking, has stimulated a tremendous amount of research in the fields of neuroscience, neuroanatomy and more recently connectomics. The diffusion tensor model was pivotal in getting these approaches established and widely adopted. Nonetheless, it is now clear that the nature of brain white matter requires more complex approaches to properly map multiple fibre orientations. Many such approaches have been proposed, and many are widely and freely available.

Methods such as spherical deconvolution provide a direct estimate of the underlying distribution of fibre orientations in the living human brain and have shown to be particularly suited to tractography, and indeed most other practical and clinical uses. We therefore believe and recommend the use of fibre ODF and spherical deconvolution methods as an essential tool for the study of the human brain.
